# Professor Smith’s Reports

**Published:** 1855-09

**Authors:** 


### PROFFESSOR SMITH'S REPORTS.

PROF. J. LAWRENCE SMITH is making valuable contributions to American Science by his researches in mineralogy. His reëxamination of American minerals has corrected many false analyses, and given to the world precise and accurate information on the subject. We have before us a very able report on the "Minerals and Mineral Waters of Chili," collected by the United States Naval Astronomical Expedition, the result of his last winter's labors. To Chemists and Mineralogists the report will prove exceedingly interesting, and it must give Prof. SMITH a still higher reputation for learning, industry, and skill in those departments of science to which he is devoted. In regard to the ores brought from Chili, he says in this report, that they would lead one to believe that this country has hardly a parallel in any region of the globe for the abundance and purity of its silver and copper mines, and but for the physical difficulties connected with the surface of the country, and the scarcity of water and fuel, the wealth accruing to that government from this source would be immense. These difficulties will doubtless be overcome in time by the means which an improved art and more perfect science will supply, and these vast treasures will be made available to the world.

These labors of Prof. SMITH can never become popular. His tables of analysis will not be read, except by Chemists and Mineralogists. But they are conferring upon him an enduring name among the men who are engaged in widening the boundaries of science. And the results of his labors are interesting to all. The world owes a great debt of gratitude to analytical chemists. Silently toiling away in their laboratories, they have removed the veil which rested upon the composition of matter around us, our food, water, the atmosphere, and the earth. They have given new efficiency to art and hightened and improved all the comforts of life. They have been the great benefactors of the race. It is to them that we owe the steam-engine and the magnetic telegraph, with whatever is most characteristic of our age of advancement and progress. Let us then do honor to the patient, laborious chemist, who is noiselessly extending our knowledge of nature, enlarging the domain of art, and multiplying and heightening our domestic comforts.

